# Author Correction: Submolecular probing of the complement C5a receptor–ligand binding reveals a cooperative two-site binding mechanism

**DOI:** 10.1038/s42003-021-02174-2

**Published:** 2021-05-12

**Authors:** Andra C. Dumitru, R. N. V. Krishna Deepak, Heng Liu, Melanie Koehler, Cheng Zhang, Hao Fan, David Alsteens

**Affiliations:** 1 Université catholique de Louvain, Louvain Institute of Biomolecular Science and Technology, 1348 Louvain-la-Neuve, Belgium; 2 Bioinformatics Institute (BII), Agency for Science, Technology and Research (A*STAR), Singapore, Singapore; 3 Department of Pharmacology and Chemical Biology, School of Medicine, University of Pittsburgh, Pittsburgh, PA USA

**Keywords:** Nanoscale biophysics, Atomic force microscopy

Correction to: *Communications Biology* 10.1038/s42003-020-01518-8, published online 18 December 2020.

The original version of the Article was missing the following text from the Acknowledgements: “Figure cartoons created with BioRender.com”. The error has been corrected in the HTML and PDF versions of the Article.

