# Supplementary material for: Submolecular probing of the complement C5a receptor–ligand binding reveals a cooperative two-site binding mechanism
Source: Commun Biol. 2020 Dec 18;3:786. doi: 10.1038/s42003-020-01518-8 (PMC7749166; doi:10.1038/s42003-020-01518-8)
Supplement: Supplementary file 2 — Supplementary Information [file 42003_2020_1518_MOESM2_ESM.pdf]

Supplementary Information for

Submolecular probing of the complement C5a receptor-ligand binding reveals a cooperative two-site binding mechanism

Andra Cristina Dumitru[1#], Rama Nagesh Venkata Krishna Deepak[2#], Heng Liu[3#], Melanie Koehler[1], Cheng Zhang[3*], Hao Fan[2*], David Alsteens[1*]

[1]Université catholique de Louvain, Louvain Institute of Biomolecular Science and Technology, 1348 Louvain-la-Neuve, Belgium.

[2]Bioinformatics Institute (BII), Agency for Science, Technology and Research (A*STAR), Singapore.

[3]Department of Pharmacology and Chemical Biology, School of Medicine, University of Pittsburgh, Pittsburgh, PA15261, USA.

Corresponding authors

*E-mail: david.alsteens@uclouvain.be, chengzh@pitt.edu, fanh@bii.a-star.edu.sg

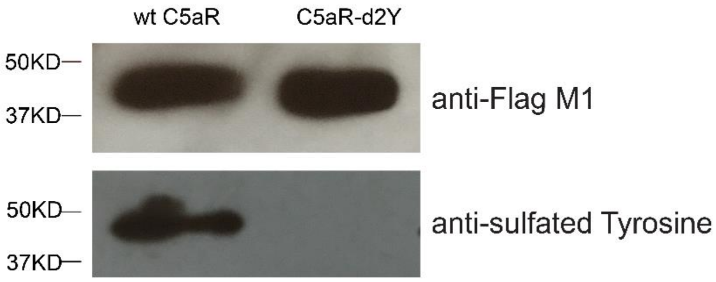

Figure S1. Western blot analysis of C5aR receptor size and sulfonation. Detection of sulfonation of wt C5aR and C5aRΔTyr with Y11F and F14F mutations (C5aR-d2Y). Both constructs are with an N-terminal FLAG tag, which was detected by an anti-Flag M1 antibody. The two mutations in C5aRΔTyr eliminated the sulfonation on C5aR. For each condition, data are representative of at least three independent experiments.

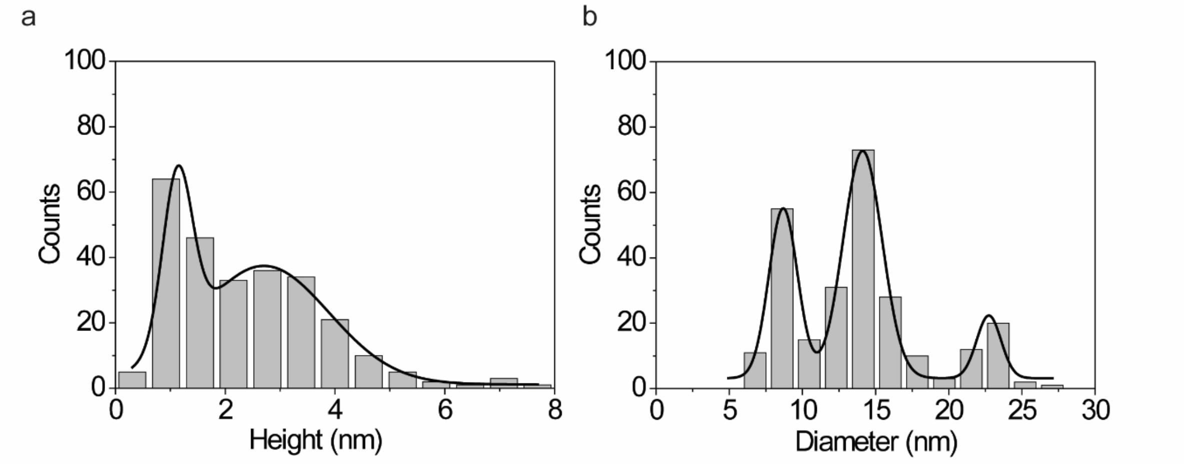

Fig. S2. Height and diameter distributions of C5aR distributed in lipid membranes. Histograms of height (**a**) and diameter (**b**) of C5aR particles imaged in **Fig 1c**. The height distribution shows two peaks centered at 1.1 ± 0.5 nm and 2.8 ± 1.2 nm. The diameter distribution shows three peaks centered at 8.6 ± 0.4 nm, 14.1 ± 0.3 nm and 23.4 ± 1.2 nm. Diameters were measured at full-width half maximum of particle heights. The number of analyzed particles in A and B is 262.

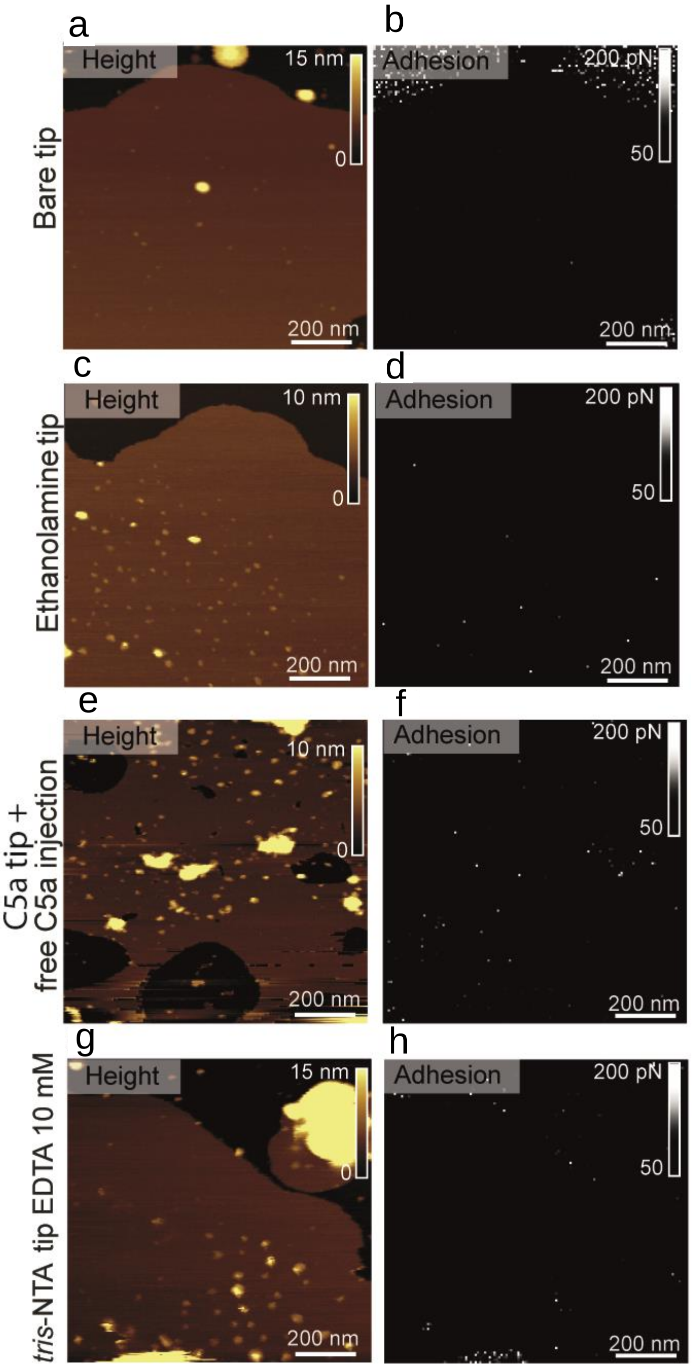

Fig. S3. Validation of adhesion events specificity measured by FD-based AFM. (a,c,e,g) AFM topography image of C5aR reconstituted in proteoliposomes and **(b, d, f, h)** corresponding adhesion map recorded either using (**a, b**) a non-functionalized AFM, (**c, d**) an ethanolamine functionalized AFM tip, (**e, f**) a C5a functionalized tip in the presence of free 1 µM C5a injected, (**g, h**) a tris-NTA functionalized tip after injection of 10 mM EDTA. All control maps only show few unspecific adhesion events. For each condition, data are representative of at least three independent experiments.

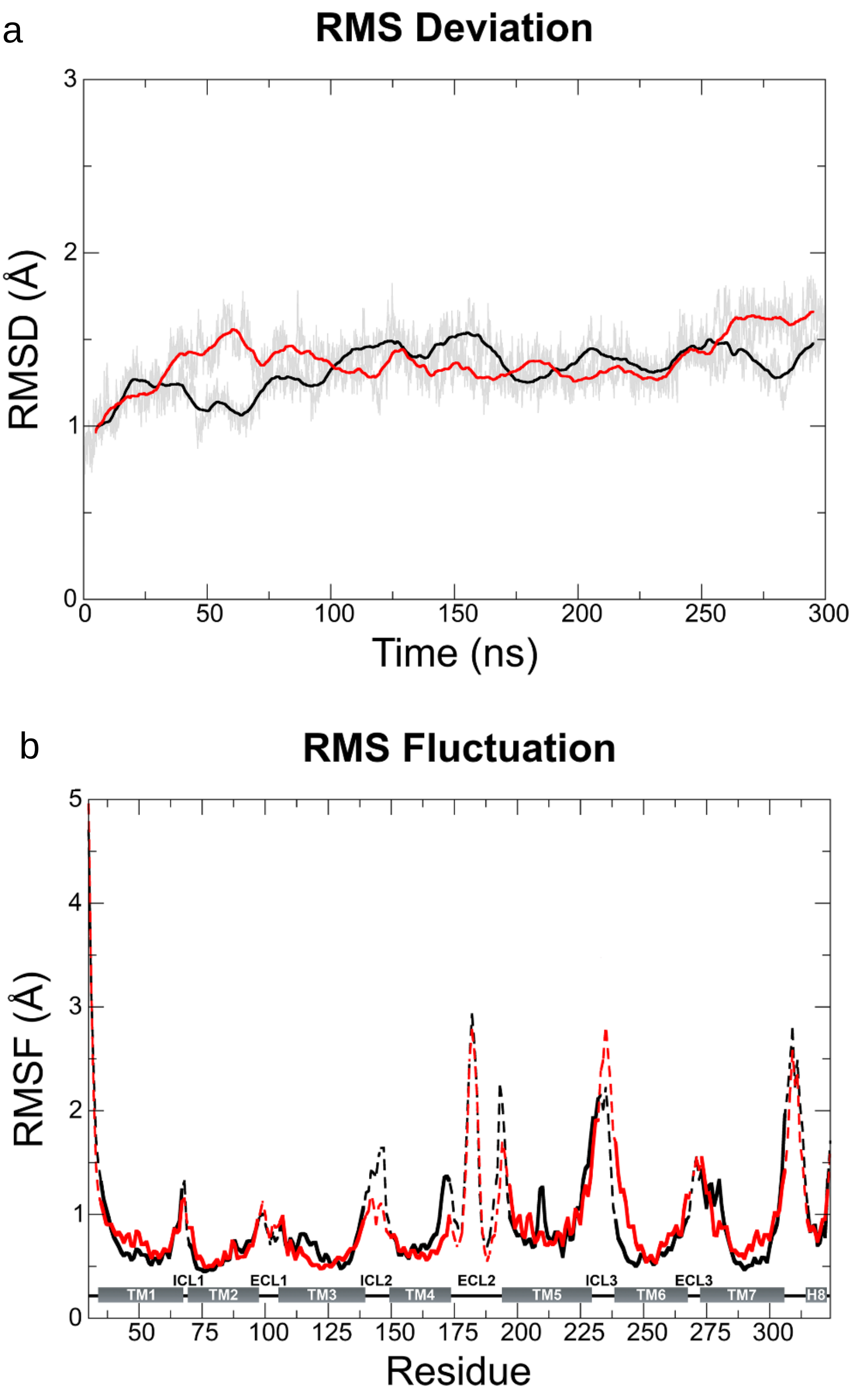

Fig. S4. Root-mean-square deviation (RMSD) and root-mean-square fluctuation (RMSF) of C5aR^{WT} and C5aR double mutant backbone atoms during 300 ns production run. (**a**) RMSD calculated for backbone atoms of C5aR-WT (black) and C5aR double mutant (red). Values are plotted at 100 ps intervals (gray) and running average was calculated over 100 frames (**b**) Residue-wise RMSF calculated for C5aR-WT (black) and C5aR double mutant (red) backbone atoms. The N-terminus, and intracellular (ICL) and extracellular loop (ECL) regions are indicated as thin dashed lines while the helical regions (TM1-7 and helix 8) as thick lines. The dashed maroon line denotes ECL2, the thick maroon lines indicate TM5 and TM7, in that order.

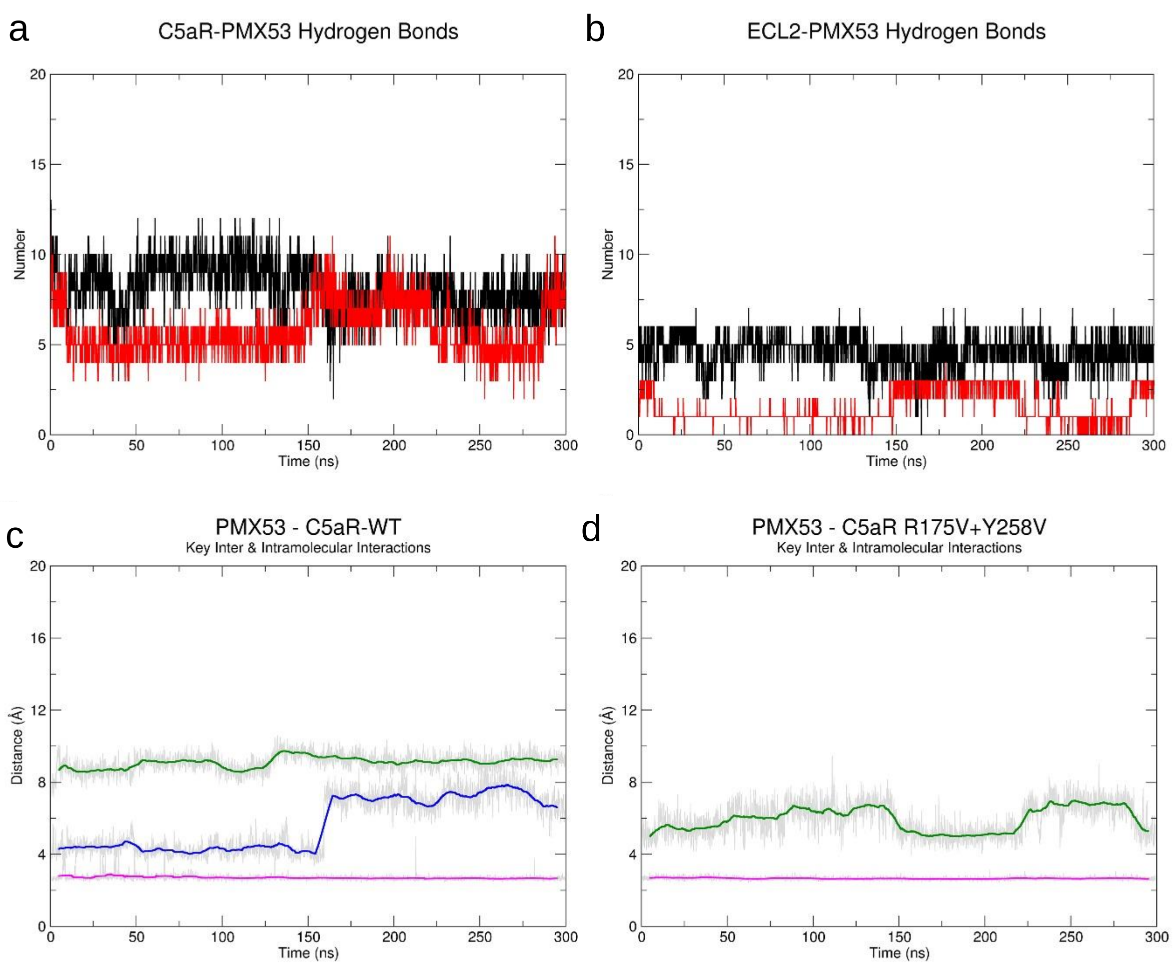

Fig. S5. Evolution of critical non-covalent interactions over the 300 ns production run in C5aR-WT and C5aR double mutant systems. (**a**) Plot of number of intramolecular hydrogen bonds formed between C5aR^{WT} and PMX53 (black), and between C5aR-double mutant (R157V+Y258V) and PMX53 (red). (**b**) Plot of number of intramolecular hydrogen bonds formed between ECL2 region (174-196) of C5aR^{WT} and PMX53 (black), and between ECL2 region of C5aR-double mutant and PMX53 (red). (**c**) Evolution of D282-R6$_{PMX53}$ salt-bridge (magenta), Y258- R6$_{PMX53}$ cation-π (blue), and R6$_{PMX53}$-W5$_{PMX53}$ cation-π (green) interactions in the C5aR-WT and PMX53 over the course of the 300 ns production run. (**d**) Evolution of D282-R6$_{PMX53}$ salt-bridge (magenta), and R6$_{PMX53}$-W5$_{PMX53}$ cation-π (green) interactions in the C5aR-double mutant and PMX53. While D282-R6$_{PMX53}$ salt-bridge remains stable throughout in both WT and double-mutant, the Y258-R6$_{PMX53}$ cation-π interaction is broken halfway through the simulation for the WT; the interaction is absent in the double-mutant. The R6$_{PMX53}$-W5$_{PMX53}$ cation-π interaction is not formed while PMX53 is bound to C5aR-WT, the interaction forms transiently in the double mutant where Y258 is mutated to V.

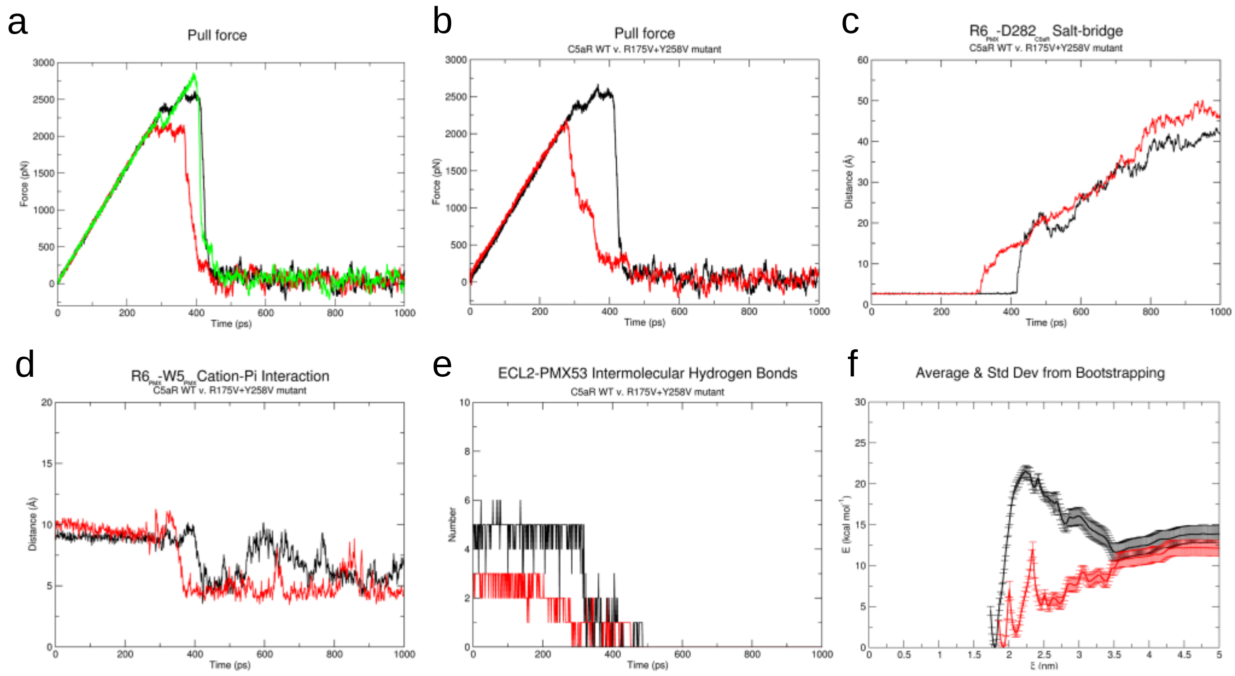

Fig. S6. SMD or COM pulling simulation of PMX53-C5aR-WT and C5aR- double mutant complexes.
(**a**) Multiple COM pulling trajectories were generated for the PMX53-C5aR^{WT} system using a pull-rate of 5 nm/ns. The trajectory that allowed for best window spacing for the subsequent umbrella sampling calculations was used, in this case the trajectory shown in black. A similar strategy was employed for the PMX53-C5aR double mutant system. (**b**) Plot showing force (pN) v. time (ps) profiles for PMX53-C5aR^{WT} (black) and PMX53-C5aR double mutant (red) derived from the COM pulling simulations used for subsequent umbrella sampling simulations. It is evident from the comparative plot that the build-up of force drops off much earlier in the double mutant system as compared to the WT. (**c**) Evolution of the $R6_{PMX53}$-$D282_{C5aR}$ salt-bridge interaction in PMX53-C5aR^{WT} (black) and PMX53-C5aR double mutant (red) derived from the COM pulling simulations. The rupture of the salt-bridge interaction in the double mutant occurs at an earlier time-point as compared to the WT, and coincides with the drop in force observed in panel (b). (**d**) Evolution of the $R6_{PMX53}$-$W5_{PMX53}$ cation… π interaction in PMX53-C5aR^{WT} (black) and PMX53-C5aR double mutant (red) derived from the COM pulling simulations. The formation of the novel interaction in the double mutant occurs at an earlier time-point as compared to the WT, and coincides with the drop in force depicted in panel (b) following the rupture of the $R6_{PMX53}$-$D282_{C5aR}$ salt-bridge depicted in panel (c). (**e**) Plot of number of intermolecular hydrogen bonds formed/broken between the ECL2 region of C5aR and PMX53 in WT (black) and double mutant (red) systems. (**F**) Average PMF profiles with standard deviation values obtained from bootstrap analysis of the PMF profiles for the PMX53-C5aR^{WT} (black) and double mutant systems.

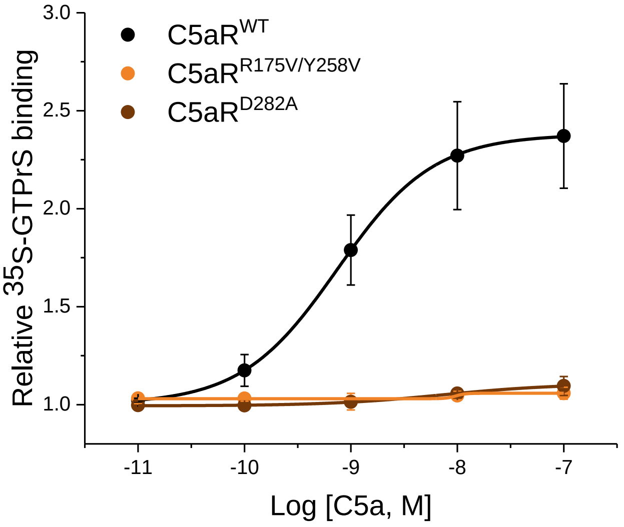

Fig. S7. Functional assays of C5aR mutants. Dose-response curves of C5a in activating G_i protein through the action on the wtC5aR and mutants. The activation of G_i protein was determined by measuring the binding of ^{35}S-GTPγS to Gi. All data are presented as mean ± SEM of 3 independent experiments.
