# Supplementary material for: Submolecular probing of the complement C5a receptor–ligand binding reveals a cooperative two-site binding mechanism
Source: Commun Biol. 2020 Dec 18;3:786. doi: 10.1038/s42003-020-01518-8 (PMC7749166; doi:10.1038/s42003-020-01518-8)
Supplement: Supplementary file 3 — Description of Supplementary Files [file 42003_2020_1518_MOESM3_ESM.pdf]

Description of Additional Supplementary Files

File name: Supplementary movie

Description: Visualization of PMX53 unbinding from C5aR facilitated by non-equilibrium centerof-mass (COM) pulling simulation method.

File name: Supplementary Data 1

Description: Source data file gathering data generated and/or analyzed in the current study, which includes those for Figs. 1d-e, 2i-k, 3h-j, 5a-d, S2a-b and S7.
